# Effects of Coronavirus-19 Induced Loneliness on Mental Health: Sleep Quality and Intolerance for Uncertainty as Mediators

**DOI:** 10.3389/fpsyt.2021.738003

**Published:** 2021-09-21

**Authors:** Simeng Gu, Zhengming He, Lianwang Sun, Yao Jiang, Minghong Xu, Guangkui Feng, Xianjun Ma, Fushun Wang, Jason H. Huang

**Affiliations:** ^1^Department of Psychology, Jiangsu University Medical School, Zhenjiang, China; ^2^Institute of Brain and Psychological Sciences, Sichuan Normal University, Chengdu, China; ^3^Department of Neurology, Lianyungang Hospital of Chinese Medicine, Affiliated Hospital of Nanjing University of Chinese Medicine, Nanjing, China; ^4^Department of Surgery, Texas A&M University College of Medicine, Temple, TX, United States

**Keywords:** intolerance for uncertainty, sleep quality, mental health, COVID-19, loneliness

## Abstract

**Objective:** The aim of the study is to investigate effects of loneliness on individual's mental health and the mediating effects of intolerance of uncertainty and sleep quality in the post Coronavirus-19 period, especially for the young people.

**Methods:** The questionnaires used in this study include UCLA loneliness scale (UCLA-3), the Pittsburgh Sleep Quality Index (PSQI), intolerance for uncertainty (IU) and the Chinese version of DASS-21. A total number of 289 subjects were recruited in the study, which includes 209 females (72.3%), 80 males (27.7%); and 212 students (73.4%), 77 working staffs (26.6%).

**Results:** The results showed that: (1) people have high levels of loneliness, anxiety, depression and stress, and poor sleep quality; (2) the mediating effect of intolerance for uncertainty in the relationship of loneliness and mental health is significant (effect size = 0.178, 95% CI confidence interval: [0.115, 0.241]), and the mediating effects of sleep quality in the relationship between loneliness and mental health is significant (effect size = 0.127, 95% CI confidence interval: [0.017, 0.239]).

**Conclusion:** Loneliness invokes a stronger self-concerned inadaptability to threat response and may lead to more mental diseases through more serious intolerance for uncertainty and insomnia.

## Introduction

The 2019 crown pneumonia epidemic (COVID-19) is characterized by rapid transmission rate and high fatality rate, and has caused huge catastrophic impacts in the world (1). In front of the ravaging epidemic, many governments have adopted measures such as maintaining social distance, restricting gatherings, home quarantining, and even sealing off the city. Under the dual pressure of the many uncertainties brought about by the epidemic and the social distancing policy adopted by the government to reduce the spread of the epidemic, the mental health during the epidemic has naturally become the focus of attention. A study on the mental health of Chinese people under the risk of COVID-19 transmission shows that most people generally have high levels of depression, anxiety, and stress (2, 3). This phenomenon is more prevalent in the city of Wuhan, which was locked down for a long tie (4). Similar findings in other countries were also reported, for example, a study by Smith et al. on the general public in the United States also shows that the global pandemic of COVID-19 and the social distancing policy adopted after the outbreak of COVID-19 have significantly increased the general public's psychological distress (depression, anxiety and stress) in the United States (5). Therefore, it is necessary to investigate the influencing factors and mechanism of people's mental health in the context of the epidemic in order to provide theoretical basis and guidance for intervention at the psychological distress.

Although current situation has improved, as one of the countries that have been affected by COVID-19 at the earliest time, the Chinese government and people have deeply suffered from the uncertainty, the strong spread and the high fatality rate of COVID-19 (6). So far, the best methods to stop the spreading COVID-19 is maintaining social distance and reducing meetings. However, the forced reduction in social interaction will have a potential impact on people's mental health (7, 8), especially loneliness (9). Loneliness is often described as the state of being without any company or in isolation from the community or society (9). It is considered to be a dark and miserable feeling, a risk factor for many mental disorders like depression, anxiety, adjustment disorder, chronic stress (10). Similar trends of increase in loneliness have been noticed among emergency workers and quarantined population in Wuhan, China (4). This has increased the prevalence of depression, anxiety, post-traumatic stress disorders and insomnia in the population (11, 12). A study focusing on mental health of children and adolescents in the context of the COVID-19 found that with the extension of isolation time, loneliness increased, thereby increasing the risk of depression, and it could predict the increase of anxiety after a period of time (13). Therefore, this study speculates that the individual's loneliness level will increase in the context of the epidemic, accompanied by higher levels of anxiety, depression, and stress.

The outbreak of the epidemic has brought out many uncertainties to people's life as well as their own health and safety, because of the high fatality of the disease. In addition, some people infected with COVID-19 are asymptomatic, it is impossible to accurately report and calculate the fatality rate, and it is impossible to know whether the people around them are infected, thus increasing the uncertainty (14). The sense of uncertainty can aggravate the individual's fear of COVID-19, causing people unable to think rationally when dealing with COVID-19, and easily induce anxiety symptoms. Uncertain Intolerance Model of generalized anxiety disorder (GAD) assumes that in the face of uncertain events, anxiety is a means of coping with discomfort (15). Intolerable Uncertainty (IU) is a kind of cognitive bias, which affects the perception, interpretation and response of individuals to uncertainty emotions at the cognitive, emotional and behavioral levels (14). Studies have found that IU can effectively predict social anxiety disorder (16), depression (17), and other psychological problems (18). Therefore, this study speculates that the loneliness will further affect the their anxiety, depression and stress levels through the individuals' paranoia toward uncertainty in the context of the epidemic.

At the end of 2020, the World Sleep Association and Zepp, a professional digital health management brand, jointly released the “*Sleep Observation in the Post-epidemic Era*,” indicating that although the social isolation policy caused an increase in individual sleep time during the epidemic, the individual sleep quality was generally not optimistic with the increase of the uncertainty and loneliness brought by the epidemic. Studies have found that the social uncertainty caused by the epidemic has disturbed people's normal sleep time, resulting in longer time to prepare for sleep and poor sleep quality, half of the people suffer from daytime dysfunction (19). Sleep quality is an important indicator for individual physical and mental health. Short-term low-quality sleep affects individual's mental state and mood, and long-term low-quality sleep affects the metabolism of brain neurons, which will lead to a series of mental illnesses. At the same time, studies have found that during the home quarantine period, people not only have a decline in sleep quality, but also have a certain degree of negative emotions, such as anxiety and depression (20); therefore, this study speculates that the individual loneliness caused by long periods of home isolation will lead to a decline in sleep quality, and at the same time further affect the individual's mental health through sleep problems.

Faced with the various uncertainties brought about by the epidemic, people's sleep quality has been severely affected, especially individuals with low tolerance for uncertainty. A study on sleep quality of adolescents in China shows that IU is related to sleep problems (21); some studies believe that uncertainty intolerance (IU) is a precursor to sleep problems (22). A study in the early stage of the COVID-19 outbreak found that intolerance of uncertainty is critical factor in the relationship between COVID-19 uncertainty and sleep outcomes (19). Based on these assumptions, this study further speculates that uncertainty intolerance can positively predict sleep quality, and play a chain-like mediating role between loneliness and mental health.

**Figure 1:**
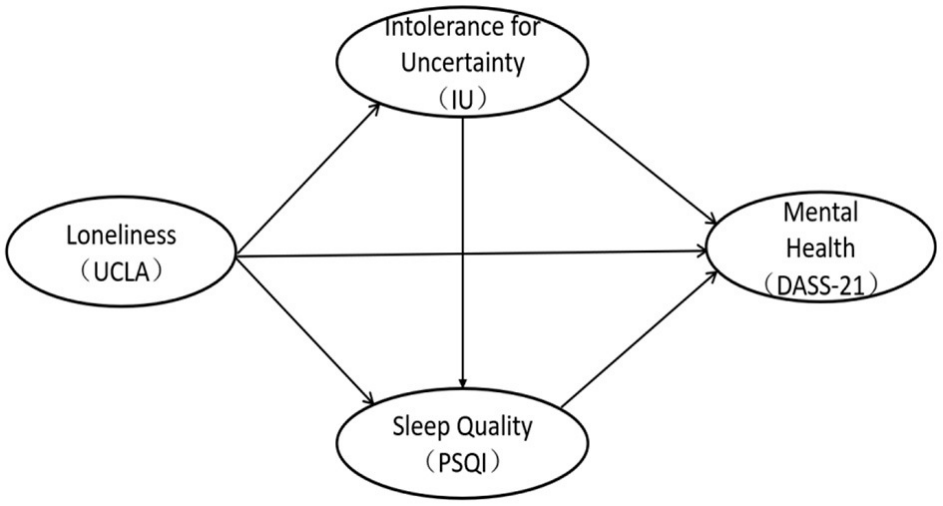
The model of the relationship between loneliness, intolerance for uncertainty, sleep quality, and mental health.

As stated in the review, this study proposes a dual mediation model of the relationship between loneliness and individual mental health (Figure 1). The specific research hypotheses are as follows. Hypothesis (1) Loneliness positively predicts the individual's depression-anxiety-stress level; hypothesis (2) Uncertainty intolerance plays a mediating role between loneliness and mental health; Hypothesis (3) Sleep quality plays a mediating role between loneliness and mental health. Hypothesis (4) Uncertainty intolerance can positively predict sleep quality, and play a chain mediating role between loneliness and mental health.

## Measures and Method

### Participants and Testing Process

A cross-sectional correlational design and a combination of convenience and snowball sampling were utilized in this study. The online survey was conducted from February to March 2021, based on Wenjuanxing platform (https://www.wjx.cn/app/survey.aspx), when local epidemics occurred in some areas, such as Hebei, Sichuan and Anhui. The survey covered most provinces of the country and collected 302 questionnaires. All of the questionnaires were initially screened by the subjects' answering time and the number of continuous answers (implemented by the R language long-string function), and 13 questionnaires were screened out, and 289 valid answers were obtained (recovery rate was 95.70%). The study was approved by the Ethics Committee of the Sichuan Normal University, and all of the participants gave their written informed consent prior to their inclusion in the study.

### Measuring Tools

#### UCLA Loneliness Scale (UCLA)

The UCLA-3 Loneliness Scale was revised and completed by Russell et al. in 1987 to evaluate the loneliness caused by the gap between the individual's desire for social interaction and the actual level (23). In this study we used the Chinese version of the UCLA-3 Loneliness Scale translated by Wang et al. (24), which includes 11 “lonely” items in positive order and 9 “non-lonely” items in reverse order. Using a 4-point score, the higher the total score, the higher the degree of loneliness. Previous studies have shown that UCLA-3 has good reliability and validity (24), and the Cronbach's α coefficient of the scale in this study is 0.91.

#### Pittsburgh Sleep Quality Index (PSQI)

The Pittsburgh Sleep Quality Index (PSQI) scale compiled by Dr. Buysse, a psychiatrist at the University of Pittsburgh in 1989, was used to assess the sleep quality of subjects in the last month (25). This study uses the Chinese version of the PSQI scale translated by Liu et al. which has good adaptability (26). The PSQI scale includes seven components, which are sleep quality, time to fall asleep, sleep time, sleep efficiency, sleep disorders, hypnotics, and daytime dysfunction. The 7 components consist of 18 items, and each component is scored on a scale of 0–3. The total score of each component is the PSQI score. The total score ranges from 0 to 21, the higher the score, the worse the quality of sleep. According to the classification standard of Liu Xianchen et al. we defined samples with a Pittsburgh sleep index greater than or equal to 8 (PSQI ≥ 8) as poor sleep quality. Previous studies have shown that PSQI has good reliability and validity (26), and the Cronbach's α coefficient of the scale in this study is 0.61.

#### Intolerance of Uncertainty Scale (IU)

The Intolerance of Uncertainty Scale (IU) was originally compiled by Freeston et al. (27) which is currently the most widely used tool for measuring uncertainty tolerance. We used the Chinese version of the IU scale compiled and revised by Huang et al. which has good applicability (28). The scale consists of 11 items with a 5-point score (1=completely non-conforming, 2=non-conforming, 3=uncertain, 4=conforming, 5=completely conforming). Higher score means lower tolerance of uncertainty. Previous studies have shown that the scale has high reliability and validity (28). The Cronbach's α coefficient of the scale in this study was 0.90.

#### Depression Anxiety Stress Scale-21 (DASS-21)

Lovibond et al. compiled The Depression Anxiety Stress (DASS) on the basis of the three-factor structural model established by Clark and Watson in 1995 to distinguish common emotional disorders such as depression, anxiety, and stress (29). DASS-21 is a simplified version of DASS, leaving the 7 projects with the highest load in each dimension. This study used the simplified Chinese version of the Depression-Anxiety-Stress Scale (DASS-21) translated by Xu et al. (30). The scale includes three dimensions of depression, anxiety, and stress, and each dimension consisted of 7 items which scored on a scale of 0–3 (0 is divided into subjects without related problems occurring within 1 week; 1 is divided into occasional occurrences, namely related problems occur once or twice a week; 2 is divided into frequent occurrence, that is, occur 3 or 4 times a week; 3 divided into always occur, that is, occur more than 5 times a week. The scale of total score span is 0–84 points. The Chinese version of DASS-21 has good reliability and validity among domestic college students, and can better reflect the level of depression, anxiety, and stress of Chinese college students (30). The Cronbach's α coefficient of the scale is 0.92.

#### Data Analysis

SPSS22.0, JASP 0.14.1.0 and Mplus8.3 were used for data analysis, with SPSS22.0 for common method deviation test and JASP 0.14.1.0 to calculate the mean and standard deviation of loneliness (UCLA), uncertainty intolerance (IU), and sleep quality (PSQI) and anxiety-depression-stress level (DASS-21), Pearson Bayesian correlation.

Mplus8.3 was used to establish a structural equation model among loneliness, uncertainty intolerance, sleep quality and individual mental health. Loneliness, uncertainty intolerance, sleep quality and individual mental health are all latent variables.

## Results

### Common Method Bias Analysis

This study uses self-reported data. Although anonymous fill-in and reverse scoring methods were used to control the test, there may still be common method deviations. Therefore, the Harman single factor test is used to perform statistical analysis on the common method deviation (31). The results showed that a total of 7 factors with characteristic roots >1 were generated, and the explanatory rate of the first common factor is 33.56%, which was less than the critical standard of 40%. It proves that there was no obvious common method deviation problem in this research, and also shows that this research can be used to construct structural equations and mediate analysis.

### Statistical Analysis of Demographic Variables

We described the demographic information of participants. The specific data are shown in Table 1. The proportion of male and female participants is about 3: 7, and the age is concentrated in 18–30 years old. The provinces where the participants lived were concentrated in Sichuan, Hebei and Anhui, where local epidemics occurred during the investigation. When investigating the occupations of the subjects, we also carried out related question options. We investigated the impact of COVID-19 on income and employment of the non-student social group, and found that 75.4% of people said that their income was not significantly affected by the epidemic, 18 % think the impact is relatively large, and 6.6% think the impact is very large. Regarding employment, 46% believe that the epidemic has no impact on employment, 33% said that the epidemic has an impact on employment, but the impact is not significant, and 18% believe that it may have an impact, and 2% believe that it has caused a significant impact.

**Table 1:** Demographic information.

**Variables**	**Number**	**Ratio %**
**Gender**		
Male	80	27.7
Female	209	72.3
**Age**		
<18	4	1.4
18–30	276	95.5
31–60	9	3.1
**Place of residence**		
Sichuan	62	21.4
Hebei	75	26.0
Anhui	59	20.4
Others	93	32.2
**Occupation**		
Students	228	78.9
Others	61	21.1
**Impact of COVID-19 on income**		
A little	46	75.4
Relatively large	11	18
Very large	4	6.6
**Impact of COVID-19 on employment**		
No effect	28	46
A little	20	33
Relatively large	11	18
Already unemployed	2	3

### Correlation Analysis

Descriptive statistics for each main variable showed that the loneliness score (*X* = 43.0) was higher than normal average score (*X* = 35.2) by 7.8 points, and the standard deviation was basically the same as the norm. It was found that 72 subjects have poor sleep quality (PSQI ≥ 8), accounting for 24.9% of the total sample. The results of the DASS-21 scale showed that the average score of DASS-21 in this study is 1 point higher than the norm.

**Figure 2:**
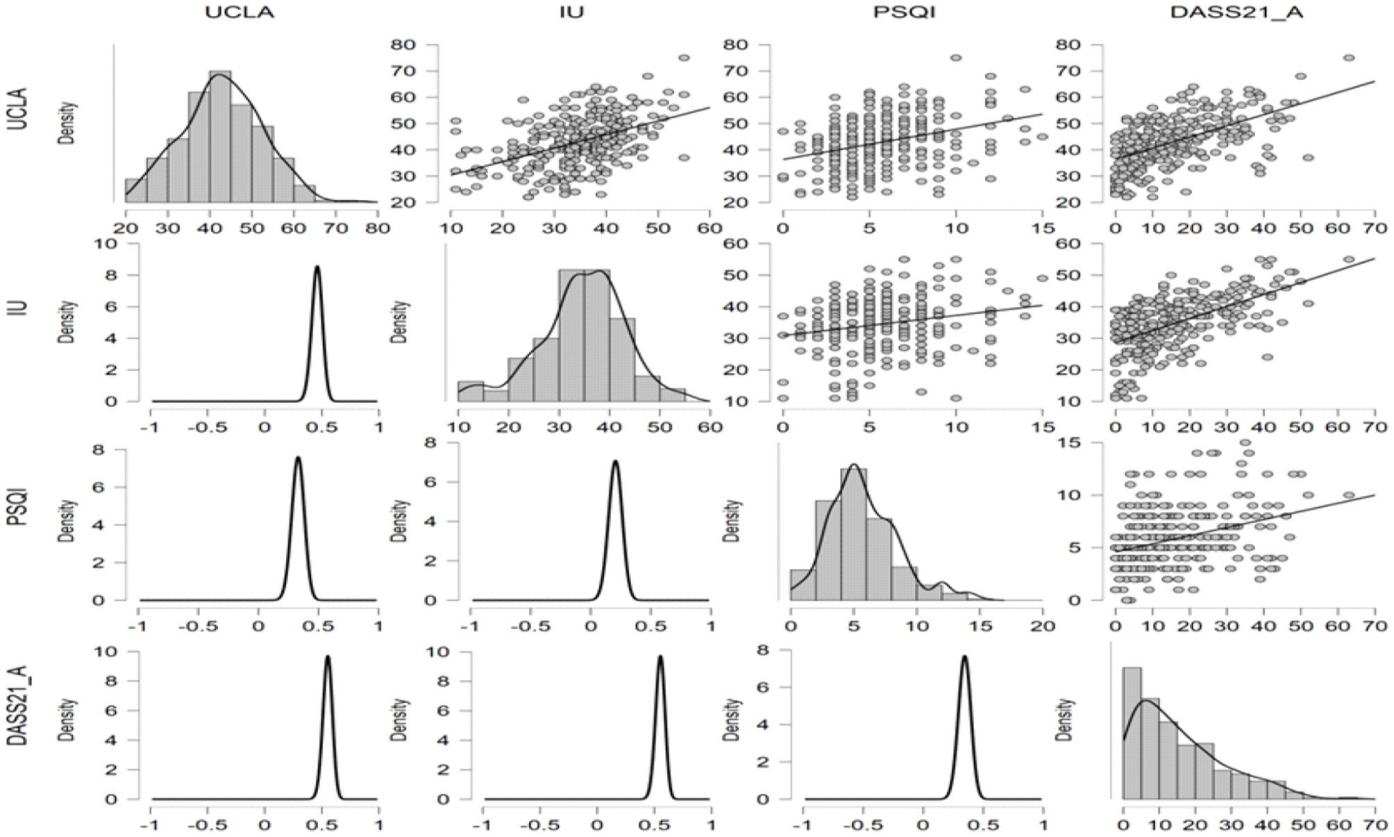
Scatter plots, density plots, and posterior probability distribution plots among variables.

**Figure 3:**
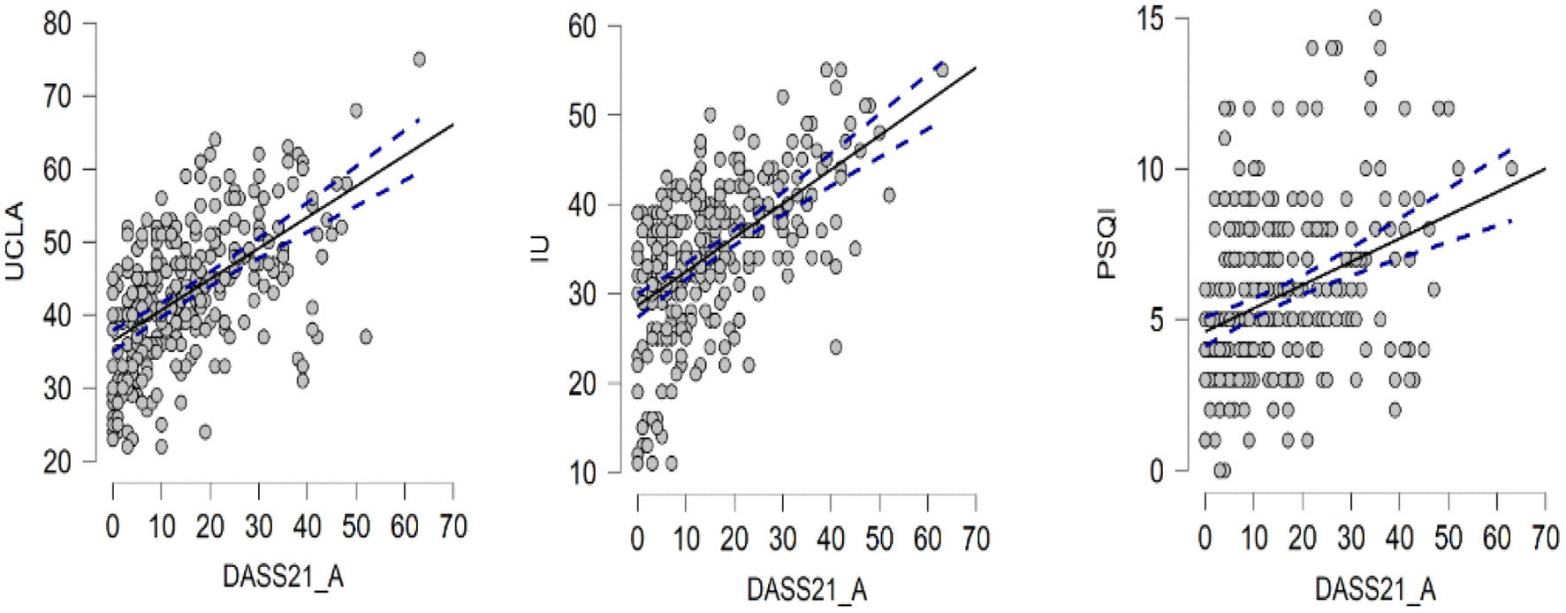
The scatter plots among UCLA, IU, PSQI, and DASS-21.

Due to the small sample size, we performed Bayesian Pearson correlation analysis on the main variables, and the results of Bayes Pearson correlation are shown in the table below. From the table, we can see that Pearson correlation among loneliness (UCLA), uncertainty intolerance (IU), and sleep quality (PSDI) and anxiety, depression, and stress level (DASS-21) scores were 0.554, 0.555, and 0.374, respectively. The correlations between the main variables and the total score of DASS-21 and the three subscales of DASS-21 were all 0.01 significant, and the Bayes factor is >100, indicating that the Bayesian statistical results absolutely support the correlation between the both. The specific results are shown in the Table 2 and Figures 2, 3.

**Table 2:** Mean, standard deviation, and Pearson Bayesian correlation matrix.

**Variable**	**Mean ± SD**		**UCLA**	**IU**	**PSQI**	**DASS21**
1. UCLA	43.0 ± 9.50	Pearson's r	—			
		BF10	—			
		Upper 95% CI	—			
		Lower 95% CI	—			
2. IU	34.5 ± 8.52	Pearson'sr	0.462^***^	—		
		BF10	5.101*e*^+13^	—		
		Upper 95% CI	0.546	—		
		Lower 95% CI	0.364	—		
3. PSQI	5.80 ± 2.76	Pearson's r	0.333^***^	0.207^**^	—	
		BF10	1.443*e*^+6^	38.313	—	
		Upper 95% CI	0.430	0.314	—	
		Lower 95% CI	0.225	0.093	—	
4. DASS21	15.5 ± 12.43	Pearson's r	0.554^***^	0.555^***^	0.347^***^	—
		BF10	3.784*e*^+21^	4.865*e*^+21^	6.465*e*^+6^	—
		Upper 95% CI	0.627	0.628	0.443	—
		Lower 95% CI	0.467	0.468	0.240	—

** *BF10 > 30,*

*** *BF10 > 100*.

### Path Analysis Results

**Figure 4:**
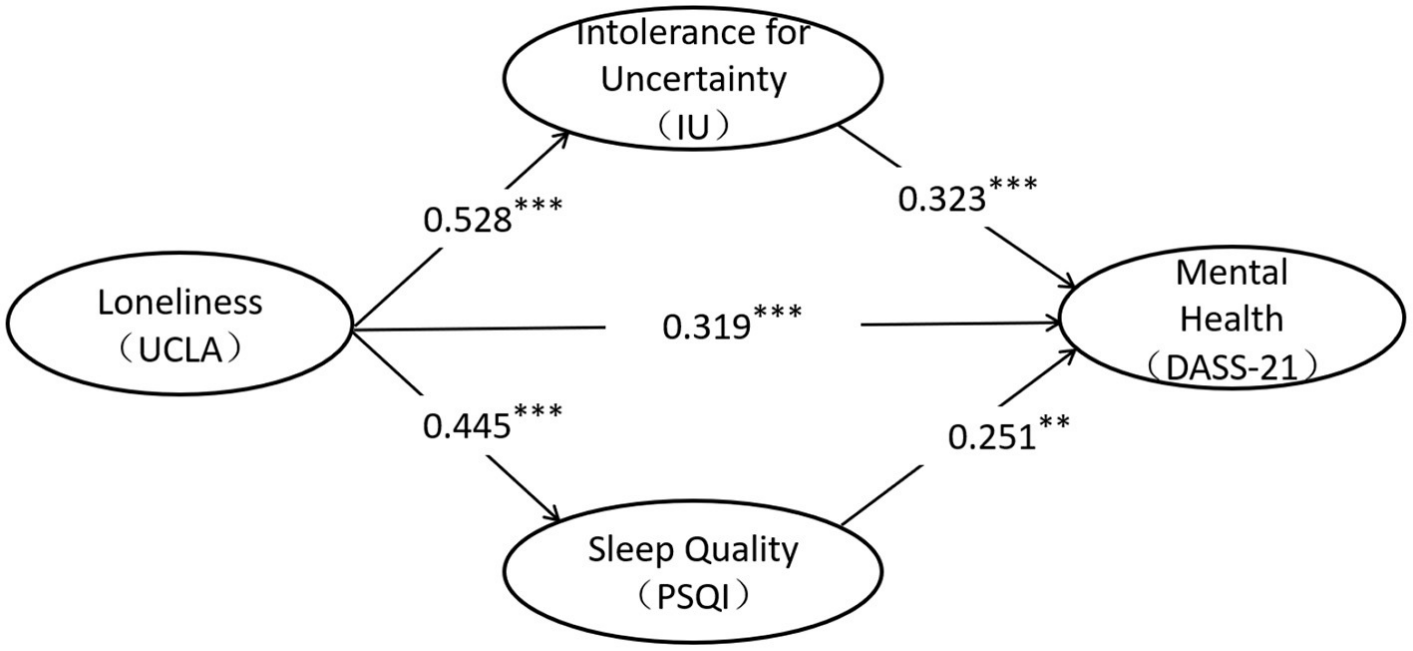
The model of the relationship between loneliness, intolerance for uncertainty, sleep quality, and mental health. **p < 0.01, **p < 0.001.

**Figure 5:**
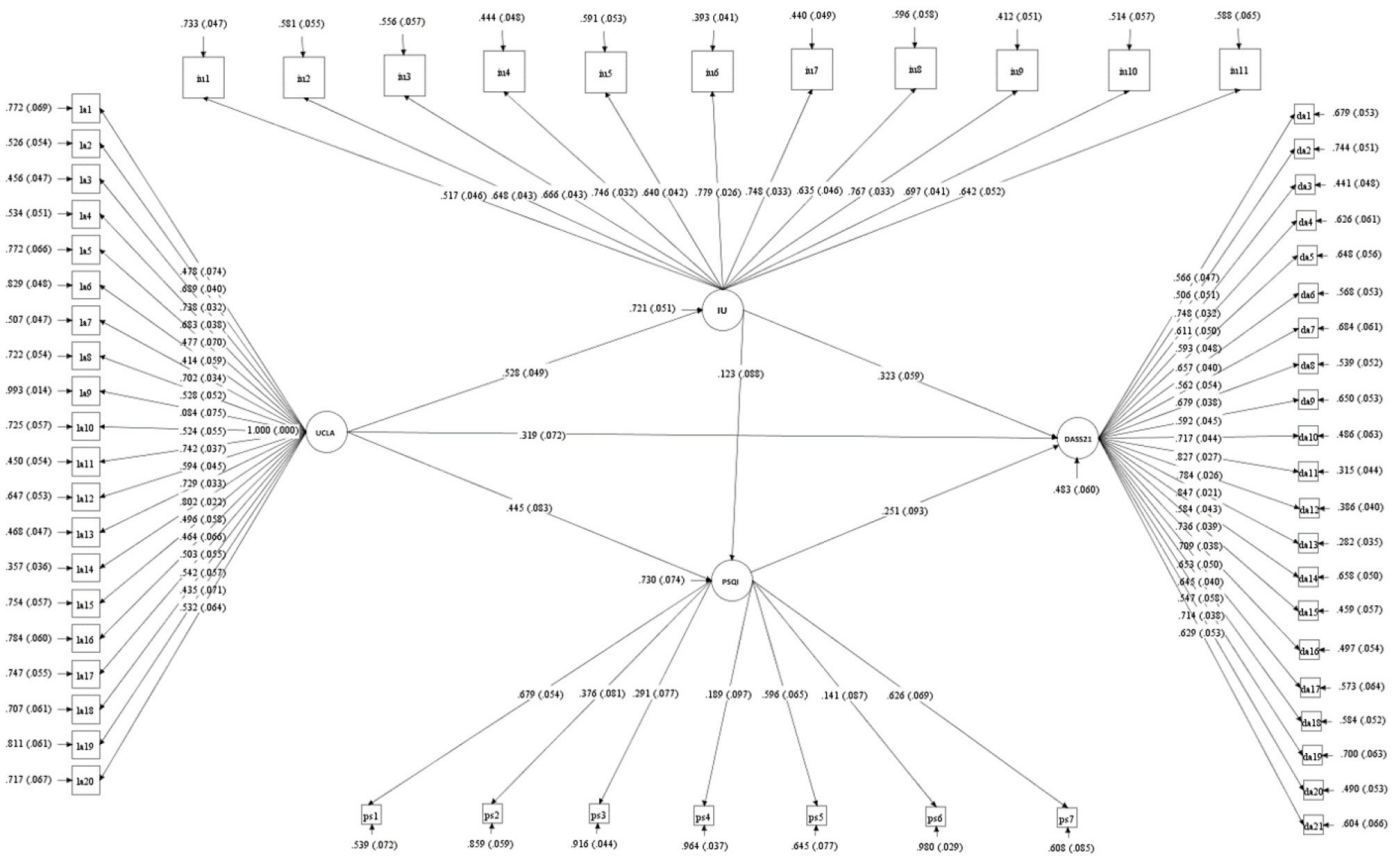
The latent variables and their corresponding explicit variables in the model. UCLA by la1-la20; IU by iu1-iu11; PSQI by ps1-ps7; DASS-21 by da1-da21.

Based on previous studies, we constructed a latent variable structural equation model of loneliness (UCLA), sleep quality (PSQI), intolerable uncertainty (IU) and individual mental health (DASS-21) (Figure 4). The details of each latent variable and its corresponding significant variables are shown in Figure 5. Among them, about the latent variable PSQI, we first encoded and scored the original data according to the statistical method of the scale (PSQI), and 18 items were composed of 7 components according to the requirements. Then, the score of each component (ps1-ps7 in Figure 5) was used as significant variables to construct the latent variable PSQI. In previous studies, researchers also used the scores of these seven components to calculate the internal consistency reliability of the scale (25, 26).

The model goodness of fit indicators are as follows: χ^2^ = 3332.367, *df* = 1646, CFI = 0.794, TLI = 0.786, SRMR = 0.069, RMSEA = 0.060, RMSEA (90% CI) = [0.057, 0.062]. According to Lai and Green's (32) summary of previous research experience, the most widely used standard for RMSEA yield the following interpretations: (a) Values < 0.05 or 0.06 suggest “good” fit; (b) values between 0.05 and 0.10 suggest “acceptable” fit. At the same time, regarding CFI, values above 0.95 are commonly considered suggesting “good” fit (32). Obviously, the value of CFI in our model is < 0.95. However, studies suggest that if the RMSEA and CFI values are inconsistent in the evaluation of model fitting, it does not necessarily represent the poor goodness of model fitting (32). Researchers should try to explain why the indices disagree. Studies suggest that in small samples (*N* <500), the estimates of the sample fit indices, mainly CFI and TLI, are likely to be biased and yield a far worse fit than their population values (33, 34). Our sample size is <500, belonging to small samples, which may be the main reason for the inconsistency of evaluation indexes.

According to the results of path analysis, loneliness affects individual's mental health through three significant paths: ① loneliness → mental health level, standardized path coefficient: 0.319^***^, hypothesis 1 holds; ② loneliness → uncertainty intolerance → mental health level, standardized path coefficient: 0.528 × 0.333 = 0.170^***^, hypothesis 2 holds; ③ loneliness → sleep quality → mental health level, standardized path coefficient: 0.445 × 0.251 = 0.112^*^, hypothesis 3 holds; ④ loneliness → sleep Quality, standardized path coefficient: 0.123; loneliness → uncertainty intolerance → sleep quality → mental health level, standardized path coefficient: 0.528 × 0.123 × 0.251 = 0.016, hypothesis 4 does not hold.

### The Mediating Effect of Sleep Quality and Intolerable Uncertainty

On the basis of model fitting, we further tested the mediating effect of uncertainty intolerance and sleep quality in the relationship between loneliness and mental health. Table 3 showed that the 95% confidence interval of the path that loneliness affecting mental health through uncertainty intolerance did not include 0. The mediating effect of uncertainty intolerance between loneliness and mental health is significant. The 95% confidence interval of loneliness affecting mental health level through sleep quality did not include 0, and the mediating effect of sleep quality between loneliness and mental health was significant. The 95% confidence interval of the path that loneliness affecting the level of mental health through uncertainty intolerance and sleep quality includes 0, and the chain mediation effect between the two levels of loneliness and mental health is not significant. In summary, loneliness can not only directly affect the individual's mental health, but also indirectly affect the individual's unbearable uncertainty and sleep quality alone.

**Table 3:** The mediating role of intolerance for uncertainty and sleep quality between loneliness and mental health.

**Path**	**Standardization** **indirect** **effect**	**Bootstrap** **(95%CI)**	**Ratio %**
UCLA → IU → DASS-21	0.170	[0.106, 0.235]	57.05
UCLA → PSQI → DASS-21	0.112	[0.012, 0.221]	37.58
UCLA → IU → PSQI → DASS-21	0.016	[−0.014, 0.046]	5.37
**Sum of indirect**	0.298	[0.190, 0.407]	100.00

## Discussion

Since the Chinese government has implemented strict and effective epidemic prevention and control policies, China's epidemic has been well controlled (6). However, due to the global spread of the epidemic, imported cases from abroad also led to unpredictable repeated epidemics in some regions of China (35). The background of this study is in January 2021. The spread of imported cases in Shijiazhuang and Chengdu led to the spread of the virus locally, and the local government immediately adopted strict epidemic prevention and control measures. At the same time, February 12 is China's most solemn traditional festival, the Spring Festival. Migrant workers working in other cities all want to go hometown and reunite with their families. However, in order to prevent and control the epidemic and reduce the flow of people, the Chinese government has proposed the call of “Don't travel as far as possible and spend the Spring Festival at current location” (36). These prevention and control measures have a great impact on people' s psychology. Studies have found that stress levels rise and happiness levels fall as a result of control measures during spring break and the uncertainty caused by the outbreak (8, 36). The survey was carried out from February to March 2021. The participants lived mainly in Sichuan, Hebei and Anhui provinces (Shijiazhuang and Chengdu belong to Hebei and Sichuan, respectively).

### The Relationship Between Loneliness and Anxiety, Depression, and Stress

Correlation analysis results showed that loneliness is significantly positively correlated with anxiety, depression and stress levels, which is consistent with previous research results (11, 37). The outbreak of the COVID-19 epidemichas seriously disturbed people's physical activity, sleep quality, and mental health. People 's negative emotions have increased and positive emotions have decreased. Negative emotions such as worry, anxiety, depression and stress have fluctuated at a high level (4, 7, 38). At the same time, the analysis of the mediation model showed that loneliness can significantly positively predict depression-anxiety-stress levels, that is, the stronger the individual's loneliness, the higher the individual's anxiety, depression, and stress levels, and the worse the mental health level (39). Previous studies have showed that when individuals are in a lonely state, their mental resilience (40) and emotional regulation ability (41) decreases, and their coping styles in the face of external events also change, often adopting negative coping styles and non-adaptive coping strategies (42). Strong psychological toughness, adaptive emotion regulation strategies and positive coping styles are protective factors for individuals to avoid anxiety, depression and relieve stress (43). Loneliness does not refer to being alone, but refers to the inner unhappiness and pain caused by the lack of some needed or desired interpersonal connections (44). Studies have shown that social support is related to the improvement of health status by reducing loneliness (45, 46). Therefore, this study enlightens us that for individuals who need to be isolated at home or concentrated during the COVID-19, relevant staff should hold an attitude of “isolated without isolating love.” While providing practical help, they should also provide psychological support to reduce individual loneliness and the risk of anxiety and depression. Similar protocols in China during the first stage of outbreak had shown to improve quality of lives of those isolated (47).

### The Mediating Role of Uncertainty Intolerance

This study found that the individual's intolerance to uncertainty (IU) plays a mediating role between loneliness and the individual's mental health during the COVID-19, that is, the higher the individual's loneliness, the more intolerable the uncertainty will be, and then the level of anxiety and depression will increase, as well as the stress sensitivity (48). Studies have found that loneliness and IU are the predictors of anxiety and depression, and loneliness is the strongest predictor, followed by IU (49). In this study, IU played a mediating role. On the one hand, it may be that loneliness during the COVID-19 makes individuals pay too much attention to themselves, triggering an unsuitable threat response; this response exacerbates the individual's intolerance to uncertainty, which causes anxiety and depression symptoms, and is more sensitive to stress. On the other hand, an individual's strategy for coping with uncertainty is usually to find social references to promote the individual's behavioral consistency with others (48), for example, the individual's behavioral manifestations are more herd behaviors. This can be seen from the crazy hoarding of daily necessities by ordinary people in various countries and the crazy spread of various rumors during the COVID-19 epidemic. However, due to the social isolation caused by the epidemic, people's social activities are restricted to a certain extent, which in turn restricts individuals with a sense of uncertainty from seeking the satisfaction of their psychological needs to be consistent with others, thereby exacerbating individuals' paranoia about uncertainty. The uncertainty intolerance model (IUM) of Generalized Anxiety Disorder (GAD) assumes that when the result is uncertain, the individual anxiety is a means of coping with discomfort (15). A study by Casale et al. after the COVID-19, epidemic showed that stressful situations increase the individual's need for social support and interaction with others; those who are usually highly concerned about their interpersonal needs will suffer more during the epidemic, especially in a social isolation environment (1).

### The Mediating Role of Sleep Quality

This study found that sleep quality plays a mediating role between loneliness and the level of individual mental health, that is, the higher the individual's loneliness, the worse the individual's sleep quality, and then their anxiety and depression levels and their stress sensitivity will increase. The poor sleep quality of lonely individuals during the COVID-19 may be caused by their excessive vigilance against potential threats in life (50), for they are unable to fall asleep peacefully or sleep deeply. Studies have indeed found that poor sleep quality caused by loneliness is an important mechanism that damages physical health (45), that is, sleep quality is not guaranteed, and leisure activities are restricted, which will seriously affect the recovery process of physical functions and damage physical and mental health. Many studies have shown that sleep disorders are closely related to individual anxiety and depression symptoms and stress perception (51, 52).

In summary, this study found that home quarantine during the COVID-19 epidemic enhanced individual loneliness; individual loneliness not only directly affects the individual's mental health, but also reduces the mental health to a certain extent through individual uncertainty intolerance and sleep disorders.

## Research Significance and Shortcomings

This study attempted to investigate the mechanism of individual uncertainty intolerance and sleep disturbance between the individual's loneliness and mental health; and the results showed that individual's uncertainty intolerance and sleep disturbance both played a certain intermediary role and established a dual intermediary model between the individual's loneliness and mental health. The results of this study enlighten us that in the process of providing social support and psychological assistance to individuals who need to stay at home or centralized isolation to reduce their loneliness and improve their mental health, it is beneficial to provide necessary psychological counseling for those. As an important treatment for psychological counseling, cognitive behavioral therapy has shown great potential in recent studies to regulate sleep quality and mental health in isolated populations (53–55). At the same time, online behavioral therapy for isolated populations has also played a huge role in recent studies (55), which can be tried to be carried out on a large scale in the future. On the one hand, relevant departments and staff should promptly publish information about the epidemic prevention and control policies and effectiveness to the public, promptly dispel rumors, and conduct scientific and orderly guidance on the work and life of the people to reduce the uncertainty caused by the epidemic; On the other hand, through a variety of online channels, people are encouraged to engage in indoor sports, to promote popular science and emotion regulation techniques and methods, such as simple mindfulness exercises, and reasonable schedules to improve sleep quality. Finally, relevant staff should investigate psychological problems, focus on individuals with anxiety and depression, who are susceptible to stress. They also need to track and monitor to prevent these individuals from aggravating anxiety.

Of course, this study also has some shortcomings. Firstly, this study is a cross-sectional study, which limits our ability to draw causal inference. However, by constructing a dual-intermediary structural equation model, we examined two indirect pathways from loneliness to mental health, which helps to understand the influencing factors and influencing mechanisms of mental health. Secondly, this study uses the individual's anxiety-depression-stress self-assessment score as an indicator of the level of mental health, and the index evaluation is not comprehensive; and this study only examined the relationship between the individual's mental quality and the level of mental health, and does not involve the differences in individual variables (such as gender and age). Finally, due to the short duration of the epidemic in some areas, large-scale questionnaires cannot be collected in a short time. The sample size of this study is not large enough, which may affect the further promotion of the results. Therefore, on the basis of expanding the sample size, follow-up studies are needed to examine the causal relationship between various psychological qualities and physical and mental health, while focusing on the investigation of group differences.

## Data Availability Statement

The raw data supporting the conclusions of this article will be made available by the authors, without undue reservation.

## Ethics Statement

The studies involving human participants were reviewed and approved by Research Project Ethical Review Application Form, Institute Of Brain And Psychological Sciences, Sichuan Normal University. The patients/participants provided their written informed consent to participate in this study.

## Author Contributions

ZH, SG, FW, and MX designed the study. ZH, LS, and YJ did the experiments, ZH, FW, GF, XM, and JH wrote the paper. All authors contributed to the article and approved the submitted version.

## Funding

The paper was supported by a grant from Foundation of Humanities and Arts from the Ministry of Education in China (19YJAZH083).

## Conflict of Interest

The authors declare that the research was conducted in the absence of any commercial or financial relationships that could be construed as a potential conflict of interest.

## Publisher's Note

All claims expressed in this article are solely those of the authors and do not necessarily represent those of their affiliated organizations, or those of the publisher, the editors and the reviewers. Any product that may be evaluated in this article, or claim that may be made by its manufacturer, is not guaranteed or endorsed by the publisher.

## References

[1] Casale S. Interpersonally-based fears during the COVID-19 pandemic: reflections on the fear of missing out and the fear of not mattering constructs. Clin Neuropsychiatry. (2020) 17:88–93. 10.36131/CN20200211 PMC8629079 34908975

[2] Cao W Fang Z Hou G Han M Xu X Dong J. The psychological impact of the COVID-19 epidemic on college students in China. Psychiatry Res. (2020) 287:112934. 10.1016/j.psychres.2020.112934 32229390 PMC7102633

[3] Wang C Pan R Wan X Tan Y Xu L McIntyre RS. A longitudinal study on the mental health of general population during the COVID-19 epidemic in China. Brain Behav Immunity. (2020) 87:40–8. 10.1016/j.bbi.2020.04.028 32298802 PMC7153528

[4] Wang C Pan R Wan X Tan Y Xu L Ho CS. Immediate psychological responses and associated factors during the initial stage of the 2019 coronavirus disease (COVID-19) epidemic among the general population in China. Int J Environ Res Public Health. (2020) 17:1729. 10.3390/ijerph17051729 32155789 PMC7084952

[5] Smith BM Twohy AJ Smith GS. Psychological inflexibility and intolerance of uncertainty moderate the relationship between social isolation and mental health outcomes during COVID-19. J Contextual Behav Sci. (2020) 18:162–74. 10.1016/j.jcbs.2020.09.005 32953435 PMC7489247

[6] Jia JS Lu X Yuan Y Xu G Jia J Christakis NA. Population flow drives spatio-temporal distribution of COVID-19 in China. Nature. (2020) 582:389–94. 10.1038/s41586-020-2284-y 32349120

[7] Brodeur A Clark AE Fleche S Powdthavee N. COVID-19, lockdowns and well-being: evidence from google trends. J Public Econ. (2021) 193:104346. 10.1016/j.jpubeco.2020.104346 33281237 PMC7703221

[8] Wang Y Wu P Liu X Li S Zhu T Zhao N. Subjective well-being of chinese sina weibo users in residential lockdown during the COVID-19 pandemic: machine learning analysis. J Med Internet Res. (2020) 22:e24775. 10.2196/24775 33290247 PMC7747794

[9] Banerjee D Rai M. Social isolation in covid-19: the impact of loneliness. J Soc Psychiatry. (2020) 66:525–7. 10.1177/0020764020922269 32349580 PMC7405628

[10] Wilson RS Krueger KR Arnold SE Schneider JA Kelly JF Barnes LL. Loneliness and risk of alzheimer disease. Arch General Psychiatry. (2007) 64:234. 10.1001/archpsyc.64.2.234 17283291

[11] Killgore WDS Cloonan SA Taylor EC Dailey NS. Loneliness: a signature mental health concern in the era of COVID-19. Psychiatry Res. (2020) 290:113117. 10.1016/j.psychres.2020.113117 32480121 PMC7255345

[12] McGinty EE Presskreischer R Han H Barry CL. Psychological distress and loneliness reported by US adults in 2018 and April 2020. JAMA. (2020) 324:93. 10.1001/jama.2020.9740 32492088 PMC7270868

[13] Loades ME Chatburn E Higson-Sweeney N Reynolds S Shafran R Brigden A. Rapid systematic review: the impact of social isolation and loneliness on the mental health of children and adolescents in the context of COVID-19. J Am Acad Child Adolescent Psychiatry. (2020) 59:1218–1239.e3. 10.1016/j.jaac.2020.05.009 32504808 PMC7267797

[14] Dugas MJ Hedayati M Karavidas A Buhr K Francis K Phillips NA. Intolerance of uncertainty and information processing: evidence of biased recall and interpretations. Cognitive Therapy Res. (2005) 29:57–70. 10.1007/s10608-005-1648-9

[15] Dugas MJ Buhr K Ladouceur R. The role of intolerance of uncertainty in etiology and maintenance. In: Heimberg RG Turk CL Mennin DS, editors. Generalized Anxiety Disorder: Advances in Research and Practice. New York, NY: The Guilford Press (2004). p. 143–63.

[16] Boelen PA Reijntjes A. Intolerance of uncertainty and social anxiety. J Anxiety Disord. (2009) 23:130–5. 10.1016/j.janxdis.2008.04.007 18565725

[17] Jong-Meyer R Beck B Riede K. Relationships between rumination, worry, intolerance of uncertainty and metacognitive beliefs. Personal Individual Diff. (2009) 46:547–51. 10.1016/j.paid.2008.12.010

[18] Dugas MJ Freeston MH Ladouceur R. Intolerance of uncertainty and problem orientation in worry. Cognitive Therapy Res. (1997) 21:593–606. 10.1023/A:102189032215322877861

[19] Wu D Yang T Hall DL Jiao G Huang L Jiao C. COVID-19 uncertainty and sleep: The roles of perceived stress and intolerance of uncertainty during the early stage of the COVID-19 outbreak. BMC Psychiatry. (2021) 21:306. 10.1186/s12888-021-03310-2 34126958 PMC8200549

[20] Marelli S Castelnuovo A Somma A Castronovo V Mombelli S Bottoni D. Impact of COVID-19 lockdown on sleep quality in university students and administration staff. J Neurol. (2021) 268:8–15. 10.1007/s00415-020-10056-6 32654065 PMC7353829

[21] Lin R-M Xie S-S Yan Y-W Yan W-J. Intolerance of uncertainty and adolescent sleep quality: the mediating role of worry. Personal Individual Diff. (2017) 108:168–73. 10.1016/j.paid.2016.12.025

[22] Lauriola M Carleton RN Tempesta D Calanna P Socci V Mosca O. A correlational analysis of the relationships among intolerance of uncertainty, anxiety sensitivity, subjective sleep quality, and insomnia symptoms. Int J Environ Res Public Health. (2019) 16:3253. 10.3390/ijerph16183253 31491841 PMC6765836

[23] Russell D Peplau LA Ferguson ML. Developing a measure of loneliness. J Personal Assessment. (1978) 42:290–4. 10.1207/s15327752jpa4203_11 660402

[24] Wang D. Reliability and validity of russell UCLA scale. Chinese J Clin Psychol. (1995) 1:23–5.

[25] Buysse DJ Reynolds CF Monk TH Berman SR Kupfer DJ. The Pittsburgh sleep quality index: a new instrument for psychiatric practice and research. Psychiatry Res. (1989) 28:193–213. 10.1016/0165-1781(89)90047-4 2748771

[26] Liu X Tang M Hu L. The correlation between sleep quality and mental health of college students. Chinese J Clin Psychol. (1995) 1:29–28+31.

[27] Freeston MH Rhéaume J Letarte H Dugas MJ Ladouceur R. Why do people worry. Pers Individ Dif. (1994) 17:791–802. 10.1016/0191-8869(94)90048-5

[28] Huang R Li J Li W. The effects of tolerance of uncertainty on risk preferences and its context-dependency. J Psychol Sci. (2014) 37:1302–7. 10.1542/peds.2011-2982 23478866

[29] Lovibond PF Lovibond SH. The structure of negative emotional states: comparison of the depression anxiety stress scales (DASS) with the beck depression and anxiety inventories. Behav Res Therapy. (1995) 33:335–43. 10.1016/0005-7967(94)00075-U 7726811

[30] Long X Xie X Xu R. Psychometric properties of the chinese versions of DASS-21 in Chinese college students. Chinese J Clin Psychol. (2010) 18:443–6. 10.16128/j.cnki.1005-3611.2010.04.020

[31] Zhou H Long L. Statistical remedies for common method biases. Adv Psychol Sci. (2004) 06:942–50.

[32] Lai K Green SB. The problem with having two watches: assessment of Fit When RMSEA and CFI disagree. Multivariate Behav Res. (2016) 51:220–39. 10.1080/00273171.2015.1134306 27014948

[33] Pavlov G Maydeu-Olivares A Shi D. Using the standardized root mean squared residual (SRMR) to assess exact fit in structural equation models. Educ Psychol Measurement. (2021) 81:110–30. 10.1177/0013164420926231 33456064 PMC7797960

[34] Shi D Lee T Maydeu-Olivares A. Understanding the model size effect on SEM fit indices. Educ Psychol Measurement. (2019) 79:310–34. 10.1177/0013164418783530 30911195 PMC6425088

[35] Li Q Luo R Zhang X Meng G Dai B Liu X. Intolerance of COVID-19-related uncertainty and negative emotions among Chinese adolescents: a moderated mediation model of risk perception, social exclusion and perceived efficacy. Int J Environ Res Public Health. (2021) 18:2864. 10.3390/ijerph18062864 33799731 PMC8002157

[36] Xu G Yuan S Jia J. Survey studies of happiness under COVID-19 for people staying locally for the Chinese New Year. Nankai Business Rev. (2021) 24:204–15.

[37] McIntyre JC Worsley J Corcoran R Harrison Woods P Bentall RP. Academic and non-academic predictors of student psychological distress: the role of social identity and loneliness. J Mental Health. (2018) 27:230–9. 10.1080/09638237.2018.1437608 29436883

[38] Giuntella O Hyde K Saccardo S Sadoff S. Lifestyle and mental health disruptions during COVID-19. Proc Natl Acad Sci USA. (2021) 118:e2016632118. 10.1073/pnas.2016632118 33571107 PMC7936339

[39] Gu S Wang F Cao C Wu E Tang YY Huang JH. An integrative way for studying neural basis of basic emotions With fMRI. Front Neurosci. (2019) 13:628. 10.3389/fnins.2019.00628 31275107 PMC6593191

[40] Zhao X Zhang D Wu M Yang Y Xie H Li Y. Loneliness and depression symptoms among the elderly in nursing homes: a moderated mediation model of resilience and social support. Psychiatry Res. (2018) 268:143–51. 10.1016/j.psychres.2018.07.011 30025285

[41] Vanhalst J Luyckx K Van Petegem S Soenens B. The detrimental effects of adolescents' chronic loneliness on motivation and emotion regulation in social situations. J Youth Adolescence. (2018) 47:162–76. 10.1007/s10964-017-0686-4 28497208

[42] Zhang X Wang Y. Related research on loneliness level and coping style characteristics of college students. China J Health Psychol. (2011) 19:1008–10. 10.13342/j.cnki.cjhp.2011.08.029

[43] Gu S Wang F Patel NP Bourgeois JA Huang JH. A model for basic emotions using observations of behavior in Drosophila. Front Psychol. (2019) 10:781. 10.3389/fpsyg.2019.00781 31068849 PMC6491740

[44] Yin Q Deng G. Influence of ioneliness on psychosomatic health of college students. China J Health Psychol. (2019) 27:795–800. 10.13342/j.cnki.cjhp.2019.05.04033329134

[45] Saltzman LY Hansel TC Bordnick PS. Loneliness, isolation, and social support factors in post-COVID-19 mental health. Psychol Trauma. (2020) 12:S55–7. 10.1037/tra0000703 32551762

[46] Segrin C Domschke T. Social support, loneliness, recuperative processes, and their direct and indirect effects on health. Health Commun. (2011) 26:221–32. 10.1080/10410236.2010.546771 21318918

[47] Duan L Zhu G. Psychological interventions for people affected by the COVID-19 epidemic. Lancet Psychiatry. (2020) 7:300–2. 10.1016/S2215-0366(20)30073-0 32085840 PMC7128328

[48] Gu S Li Y Liang F Feng R Zeng Z Wang F. The mediating effects of coping style on the effects of breath count mindfulness training on depressive symptoms among international students in China. Neural Plast. (2020) 2020:8859251. 10.1155/2020/8859251 32908488 PMC7474765

[49] Hill EM Hamm A. Intolerance of uncertainty, social support, and loneliness in relation to anxiety and depressive symptoms among women diagnosed with ovarian cancer. Psycho-Oncology. (2019) 28:553–60. 10.1002/pon.4975 30614141

[50] Liu Y Li H Xu X Li Y Wang Z Zhu H. The relationship between insecure attachment to depression: mediating role of sleep and cognitive reappraisal. Neural Plasticity. (2020) 2020:1931737. 10.1155/2020/1931737 32351552 PMC7178506

[51] Alvaro PK Roberts RM Harris JK. A systematic review assessing bidirectionality between disturbances, anxiety, and depression. Sleep. (2013) 36:1059–68. 10.5665/sleep.2810 23814343 PMC3669059

[52] Johnston SA Roskowski C He Z Kong L Chen W. Effects of team sports on anxiety, depression, perceived stress, and sleep quality in college students. J Am College Health. (2020) 9:1–7. 10.1080/07448481.2019.1707836 32149577

[53] Altena E Baglioni C Espie CA Ellis J Gavriloff D Holzinger B. Dealing with sleep problems during home confinement due to the COVID-19 outbreak: practical recommendations from a task force of the European CBT-I academy. J Sleep Res. (2020) 29:e13052. 10.1111/jsr.13052 32246787

[54] Cheng P Casement MD Kalmbach DA Castelan AC Drake CL. Digital cognitive behavioral therapy for insomnia promotes later health resilience during the coronavirus disease 19 (COVID-19) pandemic. Sleep. (2021) 44:zsaa258. 10.1093/sleep/zsaa258 33249492 PMC7798633

[55] Weiner L Berna F Nourry N Severac F Vidailhet P Mengin AC. Efficacy of an online cognitive behavioral therapy program developed for healthcare workers during the COVID-19 pandemic: the REduction of STress (REST) study protocol for a randomized controlled trial. Trials. (2020) 21:870. 10.1186/s13063-020-04772-7 33087178 PMC7576984

